# Structural Health Monitoring Design and Performance Evaluation of a Middle-Span Bridge

**DOI:** 10.3390/s23218702

**Published:** 2023-10-25

**Authors:** Wei Xiang, Jiaoyuan Wei, Fengliang Zhang

**Affiliations:** 1 Technical Center, Shenzhen Road & Bridge Group, Shenzhen 518055, China; 2 School of Civil and Environmental Engineering, Harbin Institute of Technology, Shenzhen 518055, China; zhangfengliang@hit.edu.cn; 3 Guangdong Provincial Key Laboratory of Intelligent and Resilient Structures for Civil Engineering, Harbin Institute of Technology, Shenzhen 518055, China

**Keywords:** long-term structural health monitoring, FEM analysis, crossroad bridge, real-time analysis

## Abstract

Structural health monitoring (SHM) has attracted significant attention over the past two decades due to its ability to provide real-time insight into the condition of structures. Despite the development of several SHM systems for long-span bridges, which play a crucial role in the assessment of these structures, studies focusing on short- or middle-span bridges remain scarce. This research paper presents an efficient and practical bridge monitoring and warning system established on a middle-span bridge, a key crossroad bridge located in Shenzhen. The monitoring system consists of sensors and measuring points that collect a substantial amount of data, enabling the close monitoring of various operational indicators to facilitate the early detection of threshold exceedances. Based on this system, the subtle condition of the bridge can be evaluated, and the operational condition of the bridge can be studied through the comparative analysis of the collected data. Over four months of monitoring, data including the strain and creep of the main beam, the strain and settlement of piers and the crack width of the bridge body are observed. Furthermore, the real-time operational status of the bridge is analyzed and evaluated through the combination of the collected data and the structural finite element model.

## 1. Introduction

In the field of urban roads and bridges, compared to long-span bridges with their characteristics of long span, high cost, and long construction time, medium-span bridges within the range of 20–50 m are widely used in urban bridge design because of their flexible layout, economical application, and short construction time. As critical components of urban infrastructure systems, in-service middle-span crossroad bridges are vulnerable to inevitable deterioration due to long-term traffic demand, material aging, and harsh operational environmental effects. Continuous deterioration of middle-span crossroad bridge structures, if not repaired or reformed in a timely manner, can lead to cumulative damage, ultimately affecting structural performance to different degrees. In order to maintain the availability and integrity of existing crossroad bridges throughout their lifespan, it is essential to make sure that effective inspection and maintenance strategies are implemented in an optimal way within the limited available budgets [1].

Structural health monitoring (SHM) has been recognized as a viable technology for ensuring the structural and operational safety of infrastructure systems by providing early warning of damage, preventing costly repairs or even catastrophic failure [2]. The emergence of structural health monitoring (SHM) systems for bridges can be traced back to the 1980s. In England, a long-term monitoring instrument and automatic data acquisition system were installed on the Foyle Bridge, which primarily monitored the data of main girder deflection, temperature, strain, etc. It was the earliest monitoring system installed and was considered relatively complete [3]. In the mid to late 1980s, the United States began to install monitoring sensors on many bridges. For example, more than 500 sensors were installed on the Sunshine Skyway in Florida to measure structural parameters such as temperature, strain, and displacement during and after the bridge’s construction [4]. Muria developed a monitoring system to monitor the dynamic characteristics of a Tampico cable-stayed bridge with a total length of 1543 m [5]. With the rapid development of signal processing, sensing, data collection, and computing technology in recent years, the application of structural health monitoring (SHM) in engineering structures has become increasingly popular [6,7,8,9,10,11,12], and the SHM system has been increasingly implemented on different kinds of bridges, such as the Kap Shui Mun Bridge and Ting Kau Bridge [13], Runyang Bridge [14], Yonghe Bridge [15], Tsing Ma Bridge [16], and Yangtze River Bridge [17] in China, the Oresund Bridge [18], Henrique Bridge [19], and Great Belt East Bridge [20] in Europe, the I-39 Northbound Wisconsin River Bridge [21] in the US, and so on. Although many SHM systems have been established and studied, the main focus has been long-span bridges. Regarding middle-span bridges, few studies have been carried out. Compared to the intricate SHM systems employed for long-span bridges, designing an SHM system for medium-span bridges is comparatively simpler. Although it still demands higher accuracy in monitoring parameters, it requires fewer parameters and is easier to operate. Given that medium-span bridges occupy a significant proportion of urban roads, designing SHM systems for such kinds of bridges has promising prospects in practical urban road bridge applications.

In this study, based on a mid-span crossroad bridge in Shenzhen, which underwent a series of inspections and corresponding reinforcements during 2011–2017, a structural health monitoring system was designed and installed on the bridge in order to assess the effectiveness of the prior reinforcement measures and prevent the recurrence of structural defects. Section 2 mainly describes the target bridge and its existing defects. In Section 3, a detailed SHM system design for the target bridge and the determination of monitoring and warning values are presented. In Section 4, the monitoring results are mainly analyzed. Section 5 summarizes and discusses the conclusion of SHM system design and its performance.

As one of the busiest crossroad bridges in Shenzhen, the health of the target bridge is crucial not only for ensuring traffic safety on the structure but also for maintaining the safety and normal operation of the entire traffic system beneath it. Long-term continuous monitoring enables the measurement of real-time structural responses and their long-term variations, thereby enabling authorities to identify significant changes in structural behavior and take timely action [22]. In order to understand the real-time state of the bridge operation stage and prevent potential safety hazards to the traffic system on the bridge, a monitoring and warning system was established to closely monitor the bridge. As one of the most important structural response parameters, structural strain plays an important role in the SHM-based condition assessment of bridges [22,23], which allows for both the derivation of stress by structural components under in-service loadings, thereby facilitating the evaluation of the safety reserve or reliability of structural components, and the determination of other parameters like the inner forces of the monitored structural components, such as compressive or tensile forces, bending moments, shear forces, and torques [7,24,25,26,27]. Based on long-term strain monitoring data, Cardini and DeWolf developed an envelope of maximum distribution factors, peak strains, and neural axis locations to determine the structural changes of a bridge [28]. Therefore, we selected the strain of the main girder and a bridge pier as the monitoring parameters during the entire monitoring period. In addition, the displacement of the main girder and pier of the crossroad bridge as well as the crack width of the girder were also selected for long-term monitoring. The corresponding threshold was set simultaneously, which can give warnings when the indicators exceed the threshold. Based on this system, the real-time condition of the bridge can be monitored, and comparative analysis can be carried out through the analysis system, so as to grasp the running condition and ensure the safety of the bridge.

## 2. Target Bridge

**Figure 1:**
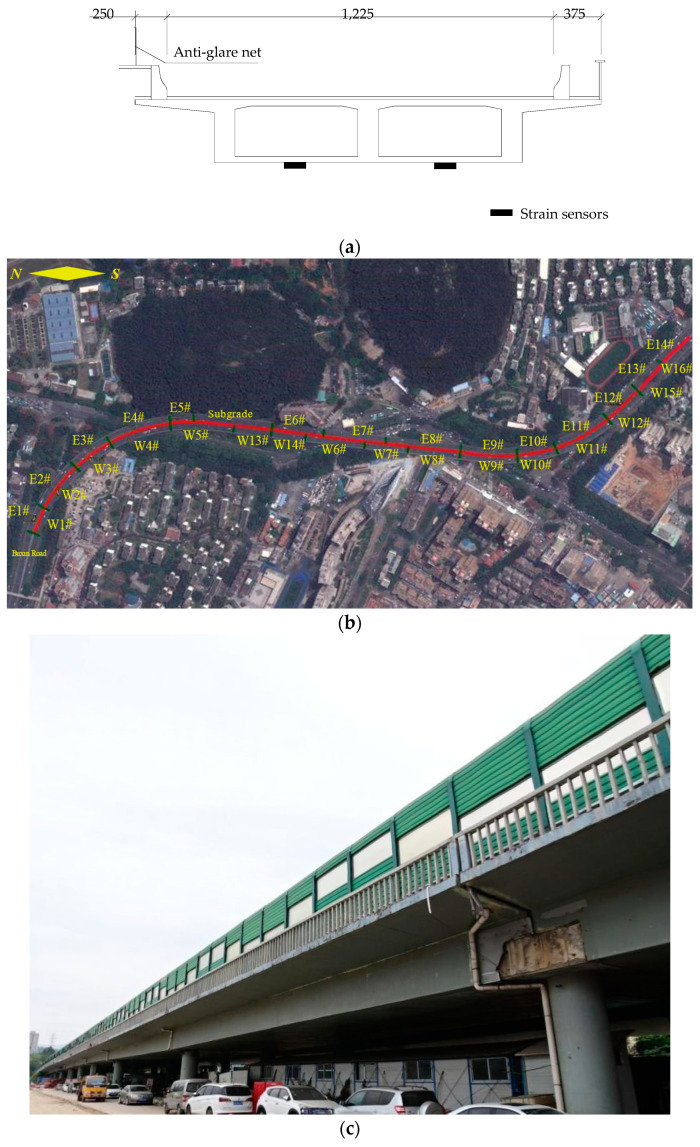
Target crossroad bridge in Shenzhen: (**a**) single-box with a double-chamber structure of the cross-section of the east bridge; (**b**) overview of the target crossroad bridge; (**c**) picture of the target crossroad bridge (underside view).

The target crossroad bridge is located in Shenzhen, China, with a total length of 1270 m along the center line of the road; it was constructed in 1999. This crossroad bridge is designed as a two-way six-lane bridge, with a superstructure prestressed continuous beam that has a common height of 2.0 m. The whole crossroad bridge is divided into two sections, the east and the west, with the east bridge comprising 14 spans, and the west bridge comprising 16 spans. The cross-section of the east bridge is single-box with a double-chamber structure (see Figure 1a) and a deck width of 14.74 m. As for the west bridge, it has the same cross-section and deck width as the east bridge, except the W13# bridge, which has a cross-section of a single box with three rooms and a deck width of 17.74 m. The piers of the crossroad bridge mainly consist of single-column piers with a diameter of 1.5 m; the top of some piers are enlarged to 2.0 m in order to accommodate bearings. Double-column piers have a diameter of 1.3 m and adopt the concealed cap beam. A picture of the target crossroad bridge is shown in Figure 1.

From 2011 to 2013, a number of bridge inspection agencies conducted inspections of the crossroad bridge and found various structural issues. The detailed results of the bridge inspection are listed as follows.

(1) In August 2011, the phenomenon of relative vertical dislocation was observed between the decks of the middle spans E16#, E17#, E18#, E21#, E23#, and E24#, as well as the corresponding parts of the west bridge, with a vertical height difference of approximately 6–9 cm.
(2) In November 2012, bridges 2#, 3#, and 4# were found to be displaced to the outside of the curve to varying degrees, with bridge 3# being the most serious, with an average value of 8.0 cm and a maximum value of 10.0 cm. At the same time, varying degrees of cracking were found in the webs and bottom plates of prestressed concrete box girders in the east and west bridges 2#, 3#, and 4#.
(3) In June 2013, it was observed that bridge 3# in the east had a tendency of creeping to the outside again.
(4) In August 2013, an emergency inspection of the bridge was conducted, and the inspection results showed that the bridge had the following main issues:
  (a) The results of the beam inspection indicated that vertical cracks were present in the webs of all bridges, except for the extension section of the target bridge. Furthermore, horizontal cracks were observed in the bottom plate, particularly in the curved bridge section.
  (b) Circumferential cracking was detected on the inner side of the root curve or the outer side of the upper curve of some curved-beam split piers.
  (c) The longitudinal movable support mainly exhibited the phenomenon of the upper steel plate sliding longitudinally relative to the lower steel basin.

According to the Technical Specification for Urban Bridge Maintenance (CJJ99-2003 [29]), the maintenance grade of the crossroad bridge is classified as Class I, and based on the inspection results, the bridge was evaluated as an unqualified class.

(5) In July 2014, the corresponding repair and reinforcement measures for the detected bridge faults were completed. Minor cracks measuring less than 0.2 mm were sealed with grouting, while larger cracks exceeding 0.2 mm were reinforced by wrapping steel plates. As for the lateral slip issue of the curved beam, a combination of pier beam consolidation and installation of steel plate anchor bolt limit devices was employed for reinforcement treatment.
(6) In December 2016 to January 2017, upon conducting several subsequent inspections of the bridge, it was discovered that the lateral slip problem of the curved beam, which had been a concern prior to the reinforcement work, had been effectively resolved. However it was also identified that several other problems still persisted:
  (a) The cracks in the corbel of the beam body and the circumferential cracking of the piers at the split pier of W10 and W11 spans still existed.
  (b) The newly discovered oblique cracks in the outer web of E11 and E12 span were of significant concern due to their severity.
  (c) The common pier column of the E27–W31 east-west bridge exhibited the most serious cracking, with oblique cracks present at both the root and upper portions of the column. In addition, circumferential cracks were observed to be expanding at the top of the No.1 pier of W40 in the straight section. Furthermore, 20, 12, and 8 circumferential cracks were identified in piers W34#, W36-2#, and W40-2#, respectively, with the maximum width of these cracks measuring 0.15 mm.
  (d) The cracks in the inner and outer webs of the E12# pier, which are about 4 m away from the E12# pier, had developed significantly, with the maximum width of the crack in the middle web being 1.1 mm and the maximum width of the crack in the oblique bottom slab being 0.32 mm.

**Figure 2:**
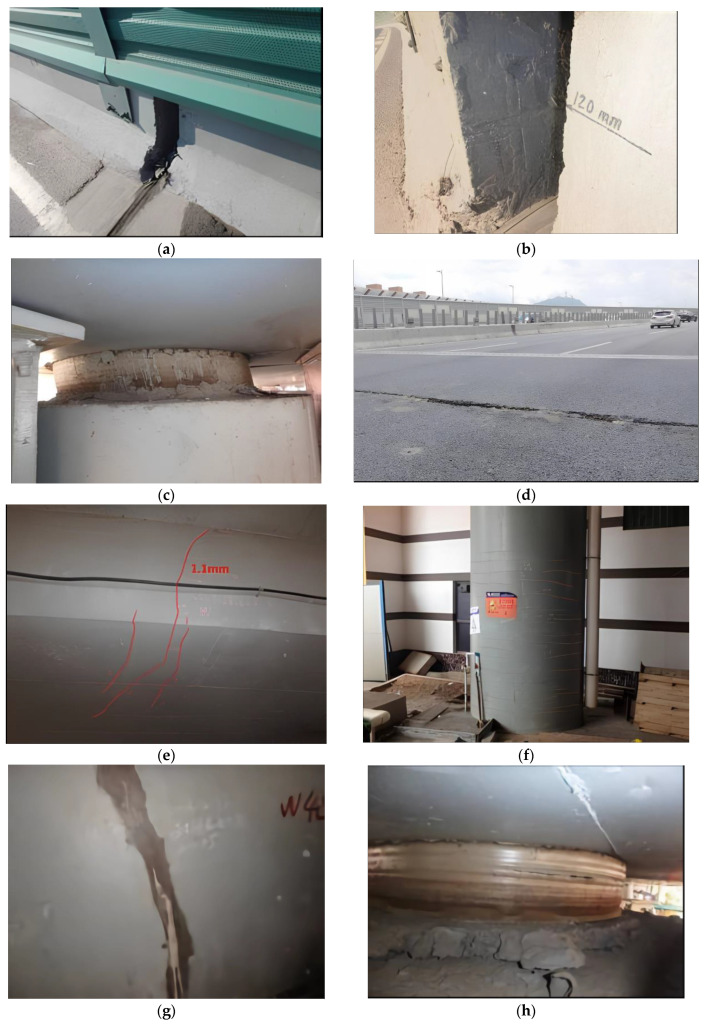
Some existing bridge defects of the target crossroad bridge before and after reinforcement: (**a**) relative vertical dislocation between the decks (2011); (**b**) lateral slip of the curved beam (2012); (**c**) shear deformation of pier support (2013); (**d**) transverse crack at the main girder approach slab (2017); (**e**) cracks found in the main girder (2017); (**f**) annular cracks in the lower part of the piers (2017); (**g**) repaired piers show new cracks (2017); (**h**) circumferential bulge deformation on pier support (2017).

In July 2014, the relevant agencies completed the reinforcement construction of the crossroad bridge, and several bridge inspections were conducted from 2016 to 2017. Based on the inspection results, it was found that the reinforcement measures for the bridge were effective, but new structural issues had emerged that were yet to be solved. Figure 2 illustrates some of the typical issues.

In order to obtain real-time information on the condition of crossroad bridges and ensure their safety, it is necessary to conduct long-term automatic monitoring and observation of the structural health status of crossroad bridges to mitigate the potential hazards associated with driving on and under crossroad bridges. Therefore, the construction of an efficient and practical monitoring and early warning system is of utmost importance in ensuring the safe operation of crossroad bridges and the highway traffic systems beneath them. Such a system must be designed to detect any potential structural issues or deterioration of crossroad bridges, thereby enabling timely maintenance and repairs in order to eliminate potential hazards associated with driving on and under crossroad bridges. By implementing such a monitoring system, the safety of both crossroad bridges and the highway traffic system can be effectively ensured.

## 3. SHM Design of the Bridge

### 3.1. Monitoring Items

**Figure 3:**
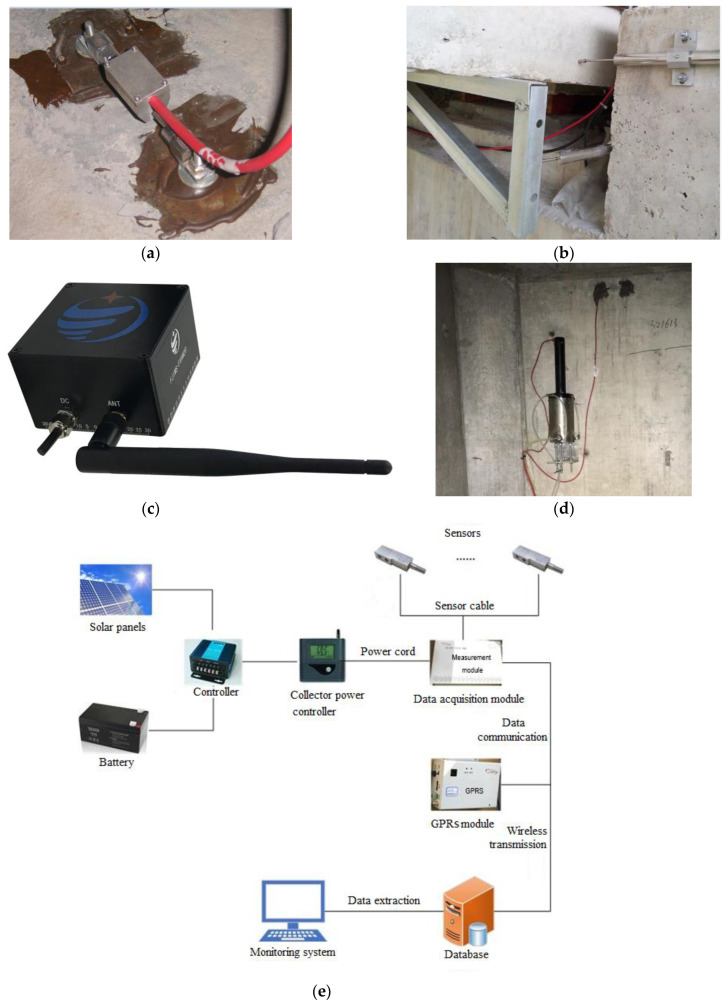
Sensors selected and schematic diagram of the monitoring items. (**a**) External vibrating string strain sensor; (**b**) magnetostrictive displacement sensor; (**c**) wireless crack-monitoring instrument; (**d**) static leveling instrument; (**e**) schematic diagram.

In order to effectively monitor the long-term health status of crossroad bridges, a comprehensive approach to structural response analysis must be employed. According to the characteristics of this type of bridge and the actual situation, five aspects of the structural response are monitored [30]:

(1) Strain monitoring of the main girder: strain sensors are installed to monitor the strain of critical sections and high-risk points of the main girder (See Figure 3a).
(2) Bridge pier strain monitoring: strain sensors are installed to monitor the strain (stress) of key sections of bridge piers (See Figure 3a).
(3) Monitoring of main beam creep: the magnetostrictive displacement sensors are installed to monitor the relative displacement between the beam body and the pier structure (including the status of supports and limiting devices) in the area where main beam creep may occur (See Figure 3b).
(4) Crack width monitoring: crack width changes are monitored using a seam gauge for critical cracks at the bottom of the main beam and the pier (See Figure 3c).
(5) Pier settlement monitoring: static level measurements are taken to monitor the settlement of selected piers (See Figure 3d).

For the monitoring of the strain terms, the external vibrating string strain sensor was selected, with a standard measurement range of ±1500 με, a sensitivity of 1 με, and operation within a temperature range of −20 to +80 °C; it is shown in Figure 3a. For the main beam creep monitoring, a magnetostrictive displacement sensor was selected, with a measurement range of 50 mm and temperature range of −20 to +85 °C. One end of the sensor was fixed on a pier, and the other end was in contact with the main beam by means of top contacting, as shown in Figure 3b. For the crack-monitoring items, wireless crack-monitoring instruments were applied, which were fixed on the concrete surface for de-tailed photography of structural cracks. Width image recognition was performed on the returned photos and converted to numerical values. As shown in Figure 3c, the wireless crack-monitoring instruments used have a measurement range of 6 mm, with an accuracy of 0.01 mm and a temperature range of −5 to +55 °C. All three types of sensors selected can measure the temperature of the measuring point synchronously. For the measurement of pier settlement, static leveling instruments were used to measure the relative elevation changes between multiple points in some piers, so as to obtain pier settlement, as shown in Figure 3d. The accuracy of the static leveling instrument is 0.2 mm, with an operating temperature range from −30 °C to +80 °C.

Sampling frequency was set at once per week for crack width and pier settlement monitoring, and for other monitoring it was set at once per hour. All sensor data were collected through the data acquisition devices, then packaged and transmitted via a wireless network to the remote bridge-monitoring center and managed and analyzed in real time on computer terminals. The schematic diagram of the whole monitoring system is shown in Figure 3e.

### 3.2. Measuring-Point Arrangement

#### 3.2.1. The Strain of the Main Girder

**Figure 4:**
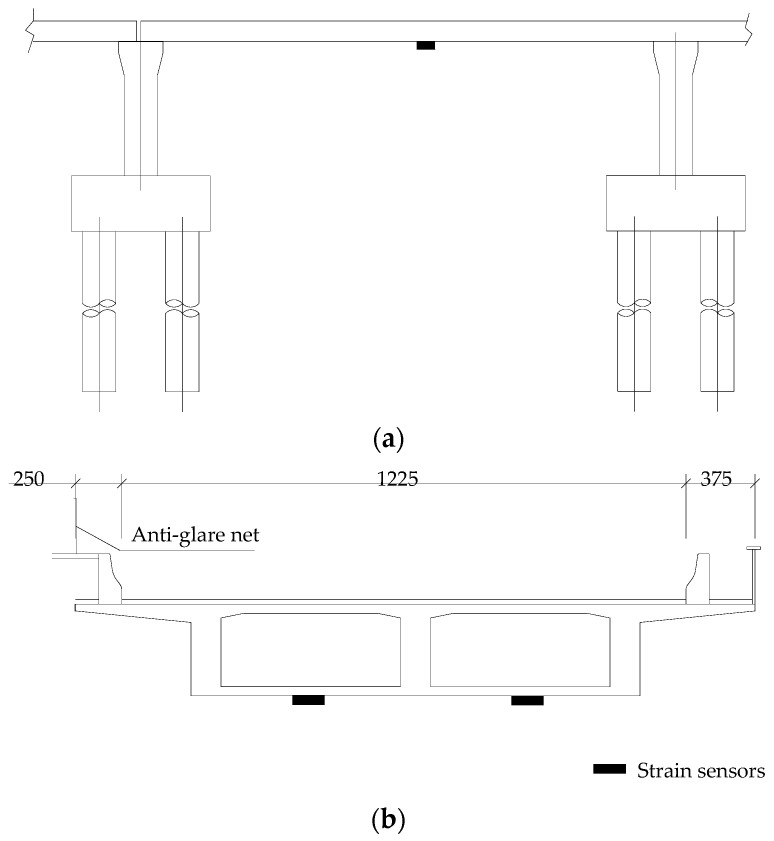
Arrangements of strain measuring points of the main girder: (**a**) longitudinal arrangement of strain measuring points; (**b**) cross-section arrangement of measuring points.

As a prestressed concrete continuous girder bridge, the strain monitoring of the main girder of the crossroad bridge focuses on the mid-span section of the main girder and the top section of a pier. The monitoring of stress (strain) levels in the main girder enables the analysis of the overall stress levels to determine if the data exceed the limit value or if stress has changed suddenly from the previous monitoring results. In light of the previous inspection results that identified vertical cracks in the webs of all bridges and horizontal cracks in the bottom plate, especially in the curved bridge section, 15 strain monitoring sections of the main girder were arranged throughout the bridge. To capture the specific defects of the bridge, with the pier top section being lightly affected, 30 measuring points were arranged in total, with two measuring points at the bottom plate of the mid-span position of the beam body selected in each section. The arrangements of strain measuring points of the main girder are shown in Figure 4.

#### 3.2.2. The Strain of Bridge Piers

**Figure 5:**
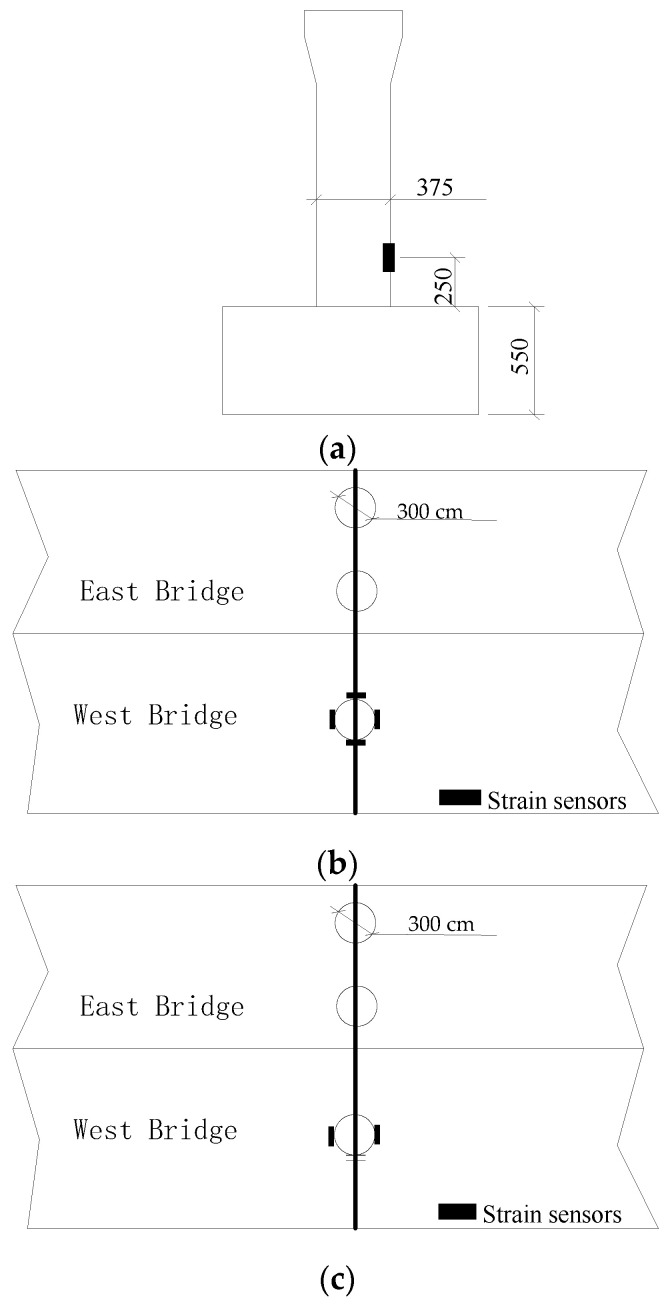
Arrangements of strain measuring points of a bridge pier: (**a**) arrangement of strain measuring points in a bridge pier; (**b**) “cross-shaped” arrangement; (**c**) “I-shaped” arrangement.

The bridge inspection report identified severe cracking in eight piers due to bridge pier defects. The most severe cracking was found in the common pier column of E27–W31 east–west bridge, with cracks at the root and upper part presenting an oblique trend. In addition, circumferential cracks were expanding at the top of No.1 pier of W40 in the straight section, with 20, 12, and 8 circumferential cracks in piers W34#, W36-2#, and W40-2#, respectively. To monitor the strain in these piers, two types of layout were used: “I-shaped” and “cross-shaped”. The “I-shaped” arrangement was distributed at two points along the radial direction of the bridge piers, and the “cross-shaped” arrangement was distributed at four points along the radial direction of the bridge piers and the radial vertical line. The measuring points were arranged 250 cm above the top surface of the pile cap, with five pier strain monitoring points arranged in a “cross-shaped” layout and three pier measuring points arranged in an “I-shaped” layout, totaling 26 measuring points. The arrangements of the strain measuring points of a bridge pier is shown in Figure 5.

#### 3.2.3. The Creep of the Main Girder

**Figure 6:**
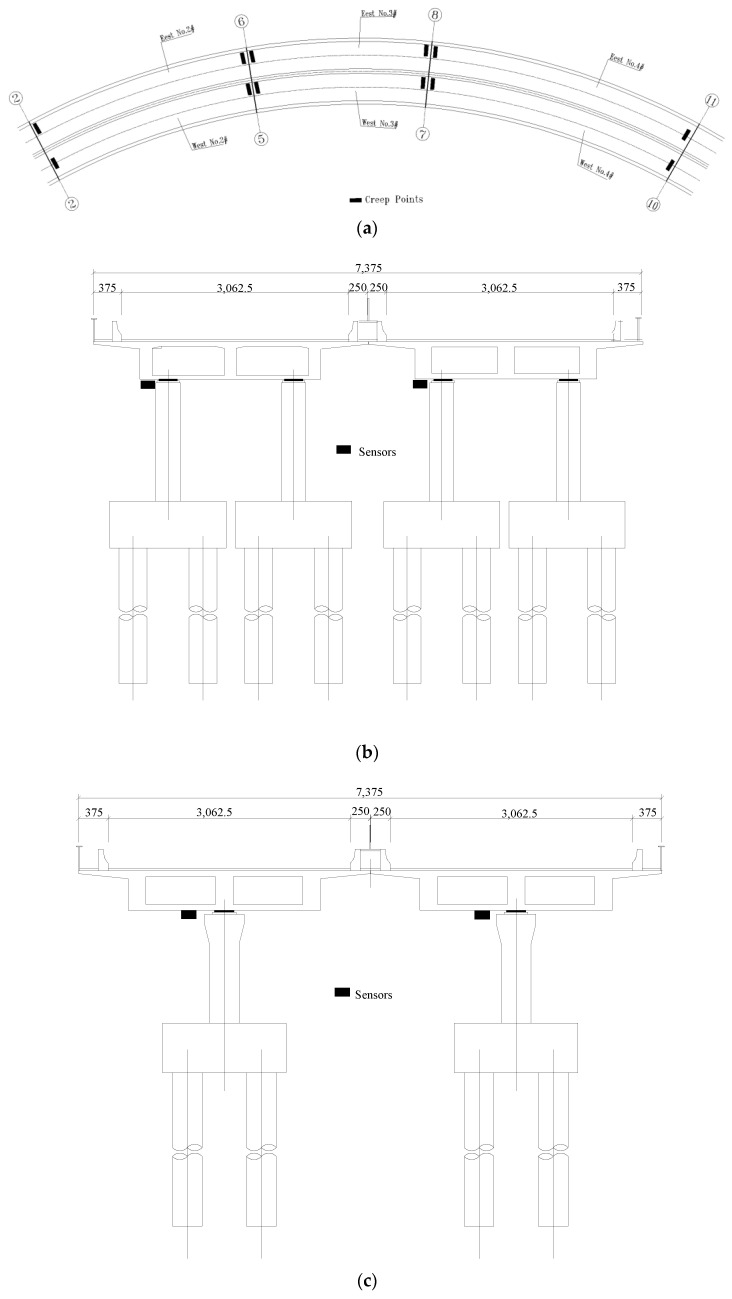

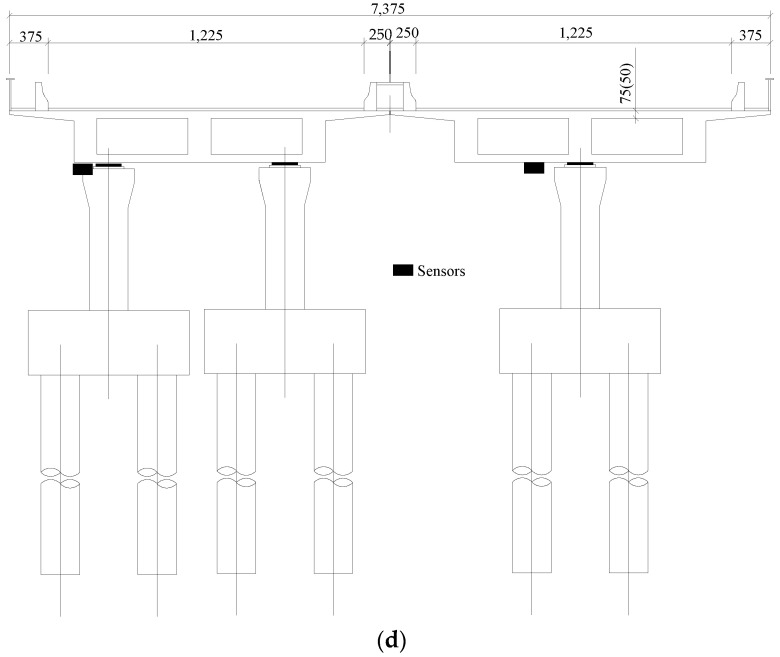
Arrangements of creep measuring points of a bridge pier: (**a**) location of creep measuring points; (**b**) cross-section I of the main girder creep measuring point; (**c**) cross-section II of the main girder creep measuring point; (**d**) cross-section III of the main girder creep measuring point.

The bridge inspection report revealed that the displacement of bridge 2#, 3#, and 4# creeps to the outside of the curve to varying degrees, with 3# in the east bridge being the most severe. To monitor the creep in these sections, creep measuring points were arranged on the main girder showing creeping. A total of 11 main girder creep sections were arranged throughout the entire bridge, with a total of 21 monitoring points, which were placed near the pier supports at the head and end of the continuous beam. The location of the shift points of a bridge pier are shown in Figure 6.

#### 3.2.4. Crack Width Monitoring

The appearance of cracks on a bridge is influenced by factors such as bridge stress characteristics, construction methods, and external environmental characteristics. The development and extension of cracks lead to the corrosion of internal reinforcement, the reduction of the stress area of the cross-section, the uneven stress of the whole beam, and the concentration of stress, which can easily cause greater damage. In the monitoring of crack width, mainly the wide, long, and extended cracks on the main girder were selected. Monitoring the development of cracks is beneficial to reveal the development law of cracks and guide later maintenance efforts. Referring to the inspection results of the bridge piers and the main girder defects in the inspection report, four crack-width measuring points were set in the whole bridge, with the crack with the largest width selected for monitoring. The specific locations are shown in Table 1.

#### 3.2.5. Settlement of Piers

**Figure 7:**
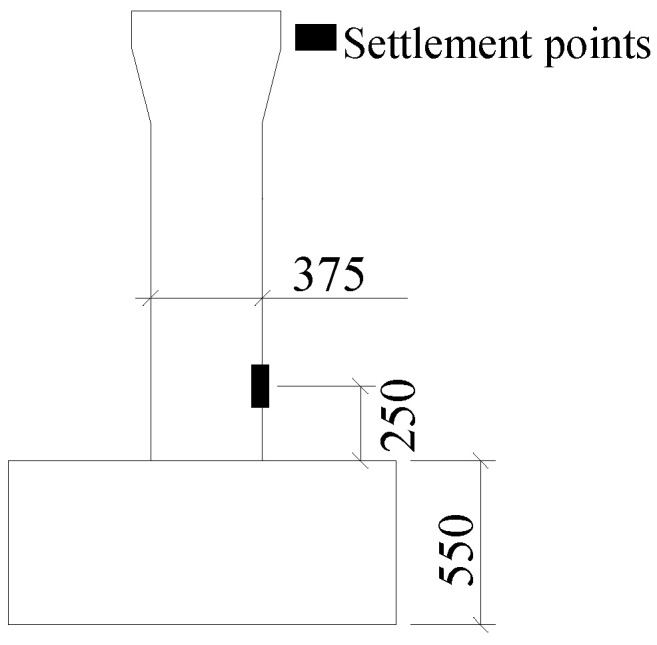
Position of settlement points of a bridge pier.

Significant construction was undertaken beneath the target crossroad bridge, including excavation of the foundation pit and underpinning of the pile foundation. Considering the distribution of bridge site construction points, seven pier settlement measuring points (including datum points) were installed across the entire bridge, positioned 25 cm above the pile caps. Data were collected on a weekly basis. The position of settlement points is shown in Table 2 and Figure 7. While determining the distribution of structural measuring points, it is also necessary to consider the corresponding early warning value to realize the abnormal early warning for the safety of the bridge. Considering the fact that the bridge has been built for a long time and has experienced structural damage and corresponding repairs and reinforcement measures, the finite element model (FEM) based on the design drawings cannot accurately simulate the actual situation of the structure. However, limited by the actual conditions of the project, the analysis results of the FEM undoubtedly still have a certain reference value for discovering abnormal data without a more accurate model. Therefore, this project used the results of finite element analysis as early warning values for reference. In the future work, some dynamic tests will be carried out to collect some vibration data, which will be used to update the FEM and make the model more accurate.

### 3.3. Finite Element Model Analysis

Based on the finite element simulation of the structure, the overall stress of the structure and the corresponding displacement and strain can be further investigated. According to the results of finite element calculation, the risk value of structural response can also be determined. Under the condition that there is no limit specified in the corresponding specification, using the finite element calculation result as the early warning value of the monitoring system can help to locate structural damage and carry out the corresponding structural repair in good time. 

**Figure 8:**
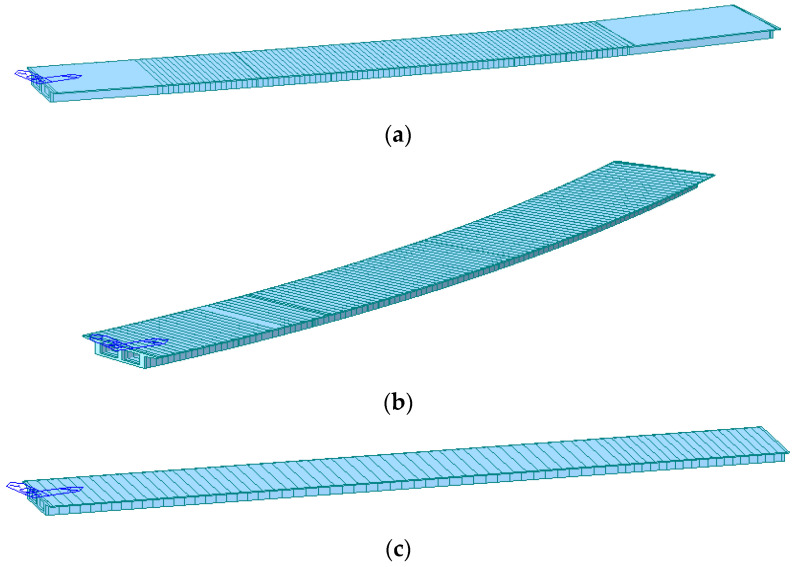
Finite element model of selected bridge sections: (**a**) West No.9#; (**b**) West No.11#; (**c**) West No.6#.

According to Midas/Civil software v8.2.1, three representative triple spans were selected to establish three bridge models, namely West No.9# (located on the transition curve and 108.199 m in length), West No.11# (located in a circular curve and 115.256 m in length), and West No.6# (located in the straight section and 112.099 m in length). Based on the design drawings, the main beams are constructed using C40 concrete, HRB335 rebar, and 1860 grade seven-wire steel strands with a diameter of 15.2 mm, of which the control stress is 1302 Mpa. In the FE model, the element type is selected as the beam element, the boundary condition is selected as the joint elastic support, the connection mode between the support and the main beam is rigid elastic connection, the live load is arranged according to the bidirectional six-lane moving load, and the internal force and deformation analysis are carried out accordingly. The completed finite element models are shown in Figure 8.

#### 3.3.1. Internal Force Analysis under Constant Load

**Figure 9:**
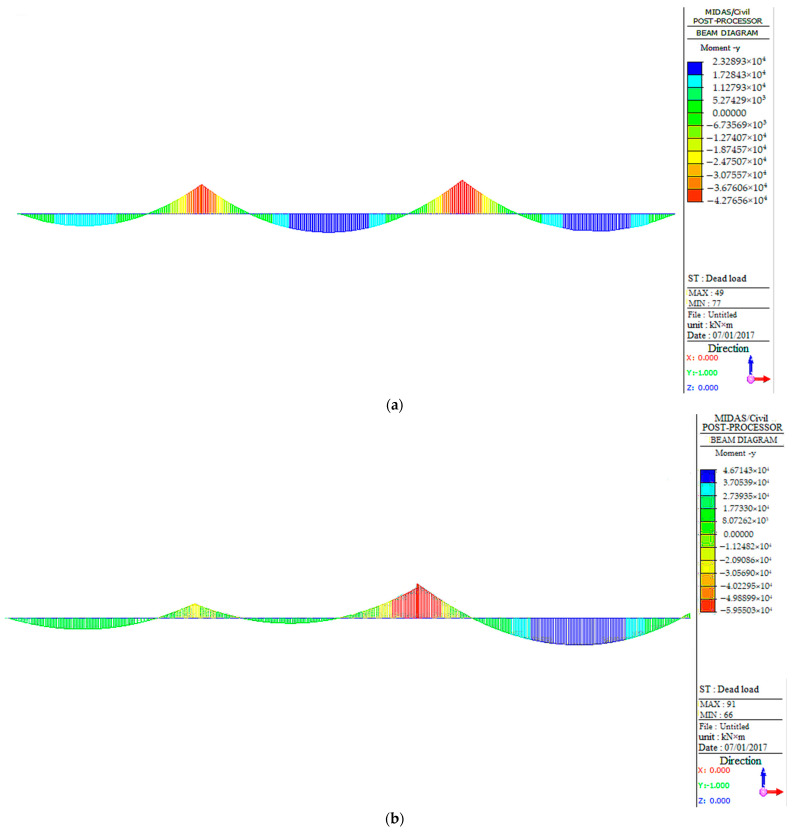

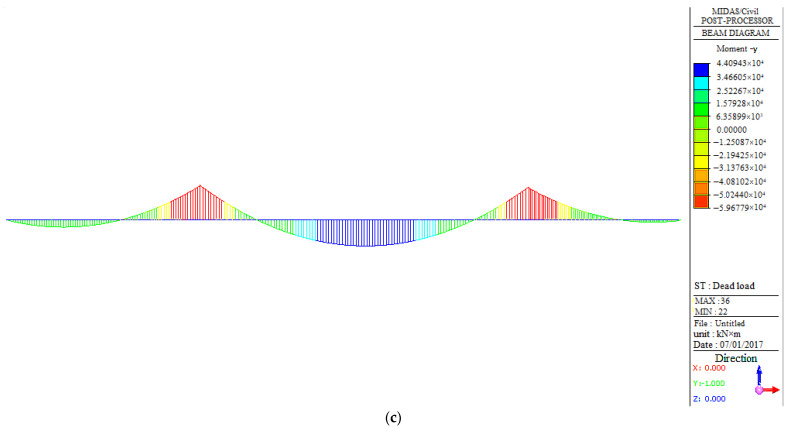
Internal force analysis under a dead load: (**a**) moment of West No.9# with a dead load; (**b**) moment of West No.11# with a dead load; (**c**) moment of West No.6# with a dead load.

The structural moment and shear force of the bridge under constant load as determined by the FEM analysis are presented in Figure 9. The maximum and minimum moments of the main girder of the triple bridge span under the action of dead load are summarized in Table 3. According to Figure 9, we can observe that under a dead load, the bending moment of West No.11# exhibits a significant asymmetric trend, with the stress on the right side being considerably greater than that on the left side; this suggests that it is crucial to pay more attention to the right mid-span bearing and deck of West No.11#. Similarly, as for West No.9#, the bending moment mainly occurs in the middle span and right span, along with the right support, which highlights the importance of monitoring the structural deformation of the middle span, right span, and right support. On the other hand, the distribution of the bending moment in West No.6# is symmetrical along the middle of the span, with an apparent trend of the middle of the span being larger than the two sides. Therefore, the focus of this section of the bridge should be primarily on the middle span and the mid-span support. In addition, Table 3 indicates that the bending moment of West No.11# and West No.6# is significantly larger than that of West No.9#, emphasizing the need to pay more attention to the structural changes of West No.11# and West No.6#.

Regarding the shear force of the bridge, it is not difficult to find that the horizontal shear forces of the three bridges are significantly small, being basically negligible. However, the shear forces of the three bridges exhibit a similar trend, with the vertical shear force being significantly greater than the horizontal shear force. It is noteworthy that the shear force on West No.9# is comparatively smaller than the others, while the other two bridges exhibit similar shear force conditions. Therefore, it is essential to focus on the shear condition of the bridges at the mid-span supports of West No.11# and West No.6#, as crucial for ensuring the structural stability and safety of the bridges.

#### 3.3.2. Internal Force Analysis under a Live Load

**Figure 10:**
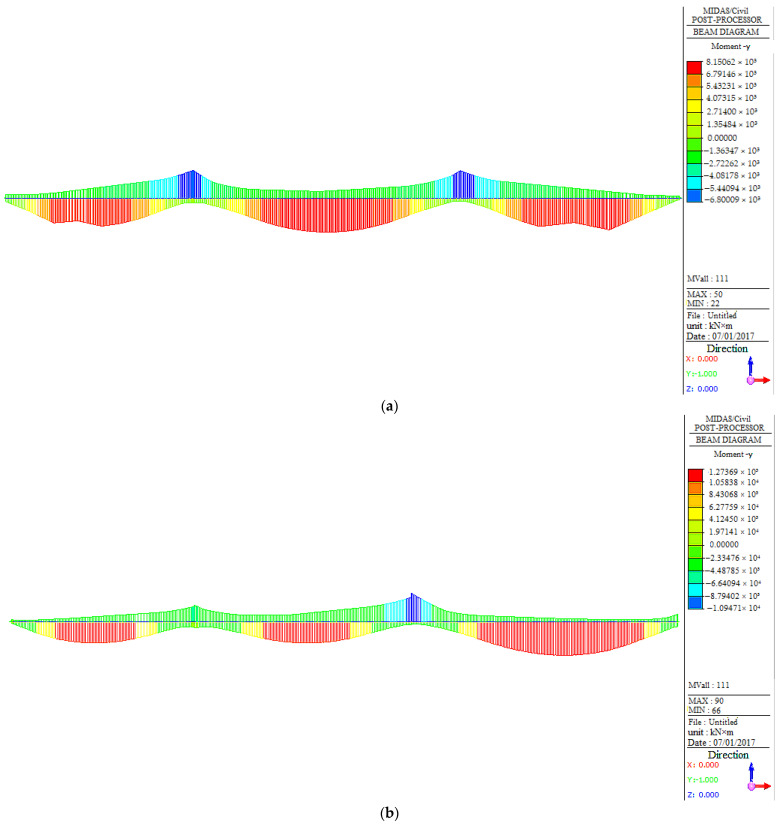

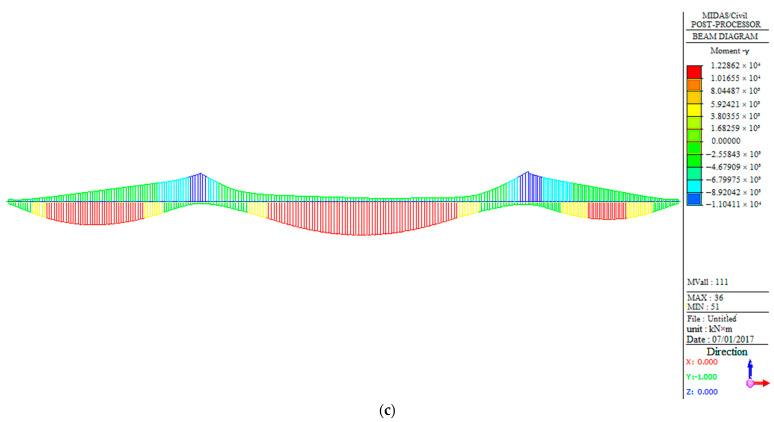
Internal force analysis under a live load: (**a**) moment of West No.9# with a live load; (**b**) moment of West No.11# with a live load; (**c**) moment of West No.6# with a live load.

**Figure 11:**
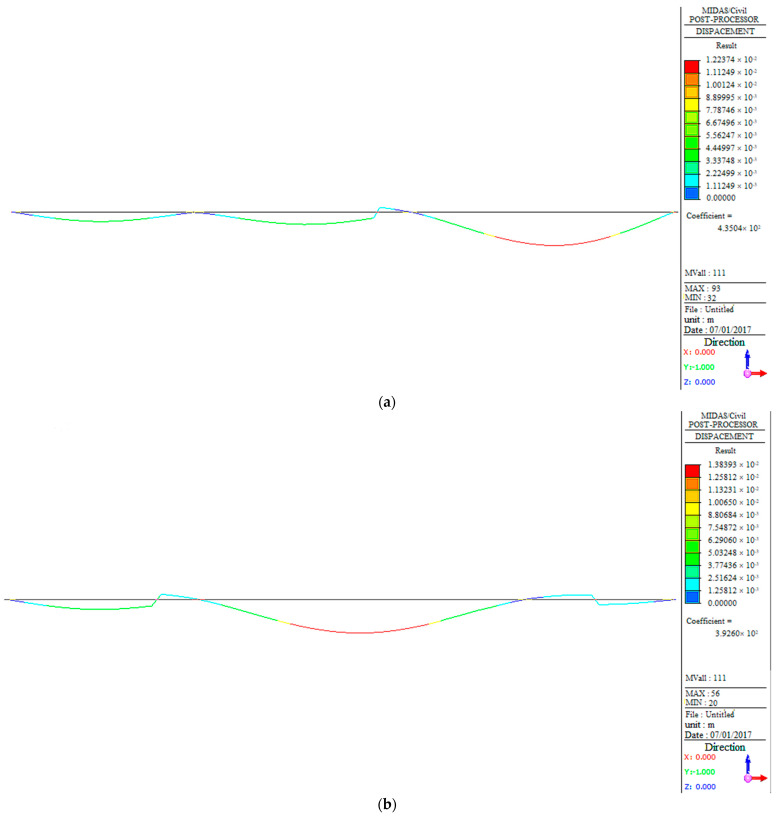

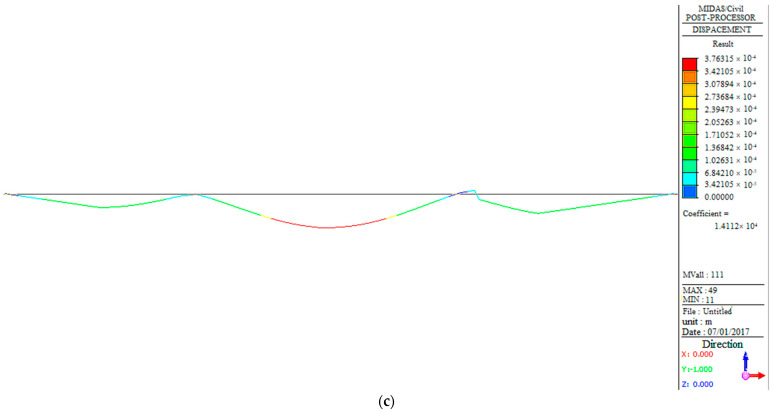
Displacement of the main girder: (**a**) deflection of West No.9#; (**b**) deflection of West No.6#; (**c**) deflection of West No.11#.

**Figure 12:**
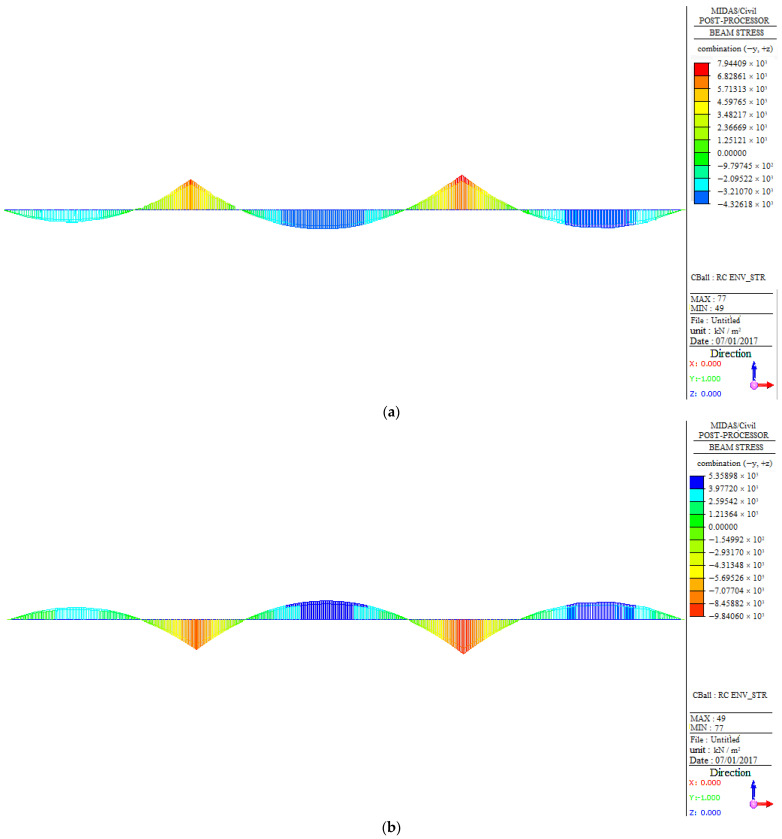

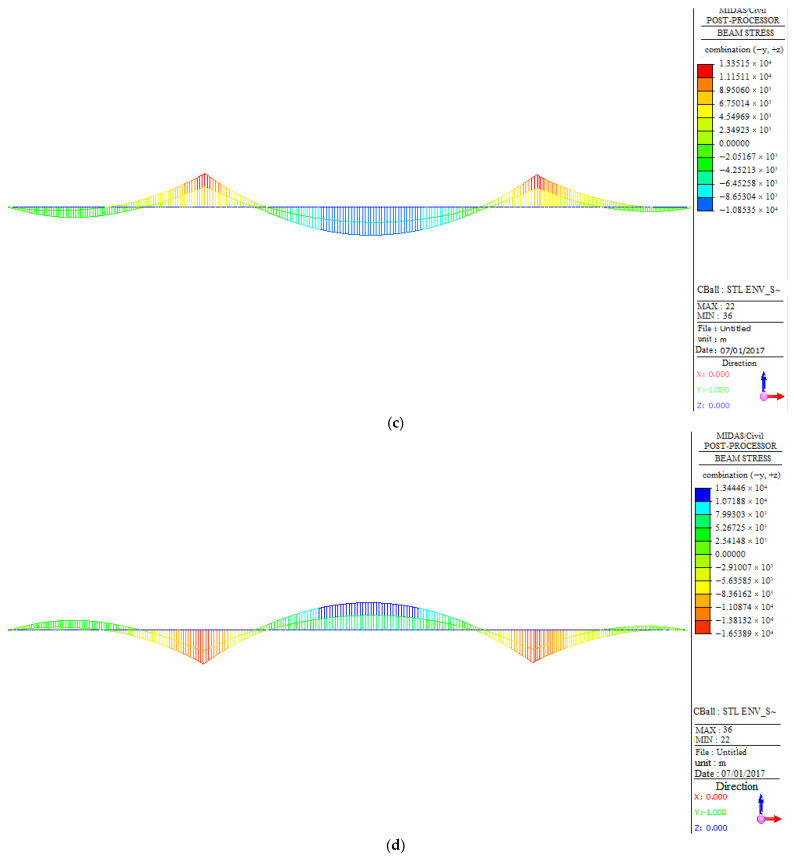

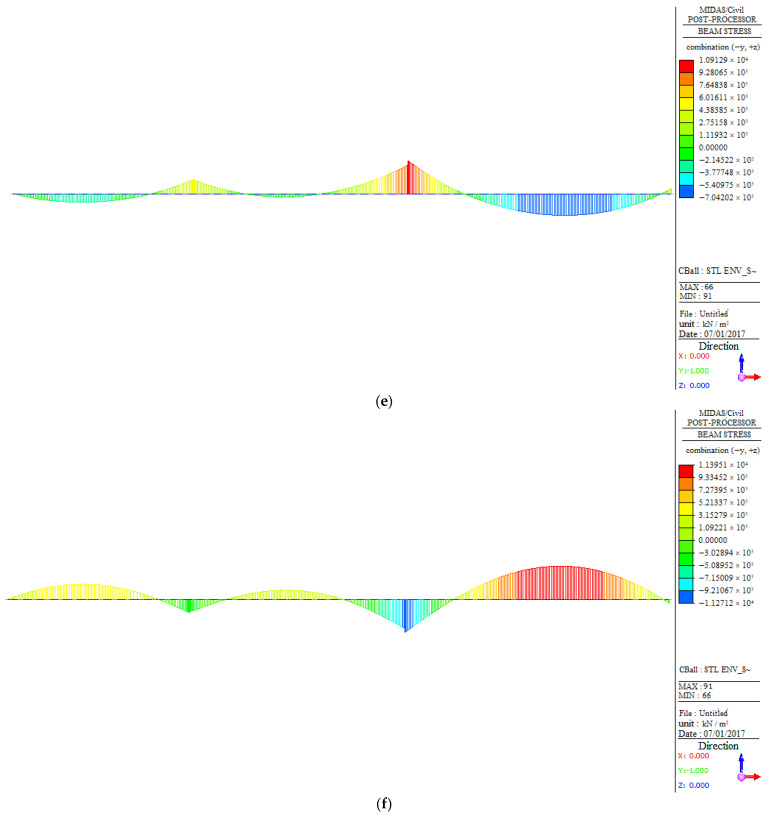
Stress distribution of the main girder under the design load: (**a**) top edge stress of West No.9#; (**b**) bottom edge stress of West No.9#; (**c**) top edge stress of West No.6#; (**d**) bottom edge stress of West No.6#; (**e**) top edge stress of West No.11#; (**f**) bottom edge stress of West No.11#.

According to the design drawings, the traffic load is transformed into a live load according to the bridge deck width of 1.5 m. This load is then distributed throughout the entire span according to the most unfavorable conditions. The envelope moment and shear envelope diagrams of the main girder under the full load of the triple bridge span are depicted in Figure 10. Table 4 summarizes the maximum and minimum moments of the main girder of the triple bridge span under a live load. The displacement of the main girder is presented in Figure 11, while the stress distribution of the main girder under the design load is illustrated in Figure 12.

Figure 10 illustrates that the structural forces under a live load vary due to the different positions of the three bridges, which is consistent with the trend observed in Figure 9. For West No.9#, the maximum positive moment appears in the middle of the mid-span, while the maximum negative moment appears in the two supports of the mid-span. Similarly, for West No.6#, the maximum positive bending moment occurs in the middle of the mid-span, and the maximum negative bending moment is located at the two supports of the mid-span. Therefore, the focus for West No.6# should be on the mid-span and its supports, while for West No.9#, attention should also be paid to the middle area of the side span. For West No.11#, the maximum positive moment appears in the middle of the right span, and the maximum negative moment is found in the right support, which means that more attention should be paid to the middle of the side span as well as to the side span supports. As for the shear force, similar to the situation under a dead load, all three bridges are subjected to vertical shear; West No.9# has the smallest shear force, while that of the other two bridges is similar.

Table 4 reveals that for the extreme moment of bridges under a dead load, the moment of West No.11# is similar to that of West No.6#, while West No.9# has the smallest moment, consistent with the moment of the bridge under a dead load (as shown in Table 3). In addition, by comparing this with Table 3, it can be found that the extreme moment under a live load is smaller than that under a dead load, suggesting that the bridges are more affected by gravity during the operation stage.

#### 3.3.3. Deflection and Stress Analysis

Furthermore, in order to monitor the deformation and stress distribution of the structure during the operation stage, the deflection and stress distribution of the structure was also analyzed based on the finite element model. Figure 11 shows that the deflection of the bridge under vehicle load follows the same trend as the bending moment diagram (Figure 10). The largest deflection for West No.9# and West No.6# occurs in the mid-span, while West No.11# has the largest deflection in the right span. Figure 12 indicates that the stress distribution of the bridge under the design load has a similar trend to that shown in Figure 10. Those parts with high structural stress should be the focus of attention, referring to the two supports and the middle position of the mid-span in West No.9# and No.6# bridges, as well as the right support of the mid-span and middle position of the right span in West No.11#.

### 3.4. Warning Values Based on FEM Analysis

The key to judging the safety state of a structural health monitoring system is to set up a reasonable early warning system. When the monitoring indicators are within the allowable range, the structure can be considered safe. When they are not, this indicates that there is a certain risk in the over-limit position of structural monitoring, which requires close consideration. Local detection should be carried out for the over-limit position to identify the reasons and carry out corresponding repairs. The design load is determined based on the General Code for Design of Highway Bridges and Culverts (JTG D60-2015 [31]), arranged according to the most unfavorable load, and the maximum calculation result corresponding to each monitoring parameter is selected as the monitoring warning value. According to the calculation of the finite element model, the maximum crack width is 0.05 mm, while for the main girder, the maximum strain is 100 με, and the maximum creep is 20 mm. As for the piers of the bridge, the maximum strain is 200 με, and the maximum settlement is 20 mm. Taking into account the results of the finite element model as well as the actual arrangement of measuring points, the warning values for each monitoring item were determined, which are summarized in Table 5.

## 4. Monitoring Results

**Figure 13:**
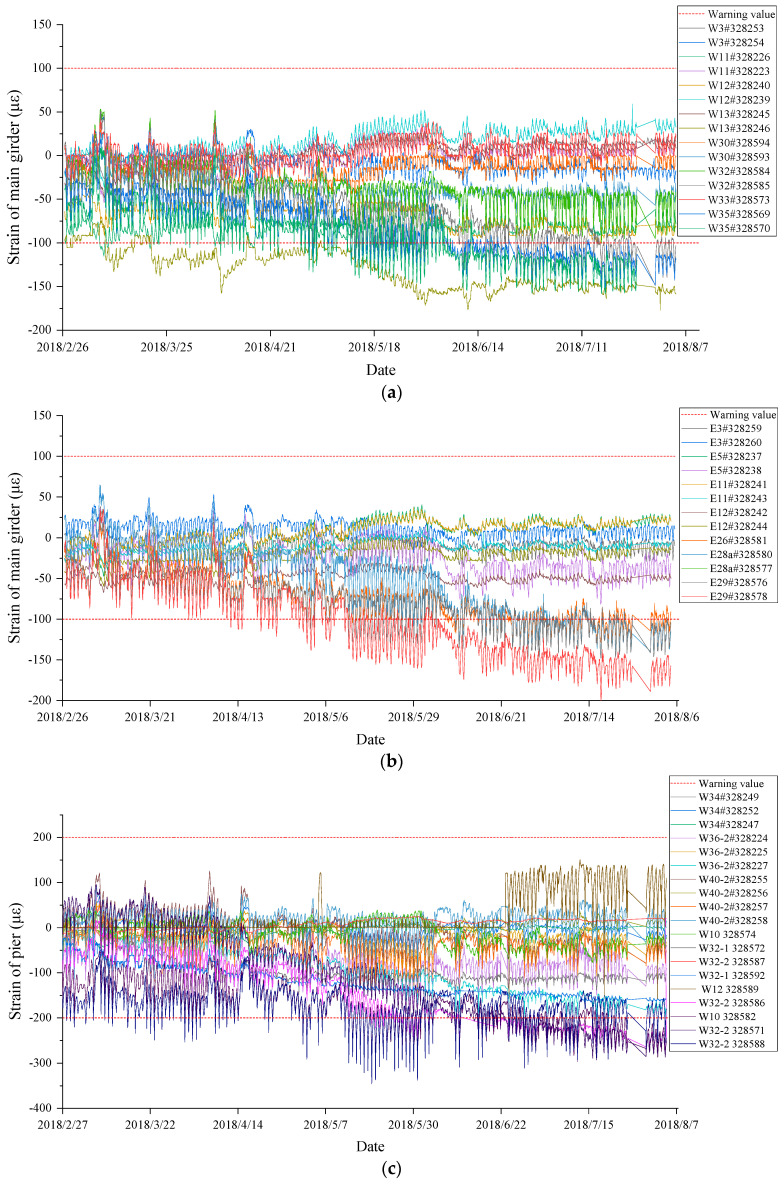

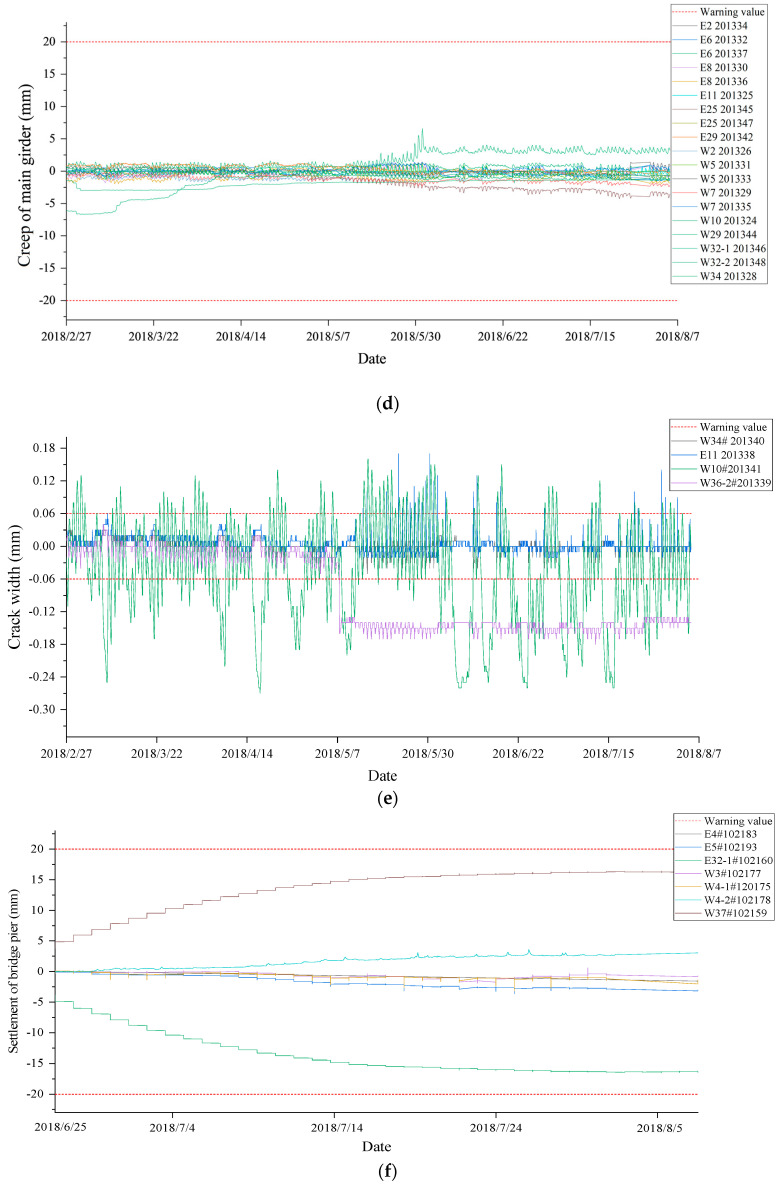
Abnormal data in time history: (**a**) strain of the main girder on west bridges; (**b**) strain of the main girder on east bridges; (**c**) strain of bridge piers; (**d**) creep of the main girder; (**e**) crack width; (**f**) settlement of bridge piers.

The monitoring process, which lasted from February to August 2018, produced data that were analyzed. They are illustrated in Figure 13, with the results summarized in Table 6. The detailed observations are listed as follows.

(1) Strain of the main girder

During the six-month monitoring period, all strain sensors of the main girder of the crossroad bridge were in normal working condition. However, the data of the measuring points at six positions exceeded the limit (listed in Table 6), which indicates that these positions require continuous attention.

(2) Strain of the bridge piers

Similarly, all of the strain-measuring sensors of the piers worked normally during the monitoring. Because some sensors were located in the construction area, the data suddenly changed obviously, but the long-term trend of the data was stable, which means that no structural defects occurred. Among the pier strain-measuring points, the data of two positions exceeded the limit (listed in Table 6), and the data of other measuring points were in a normal state. As the out-of-limit points were near the construction area under the bridge, the influence of construction on the strain of a pier should be considered, and these two positions should be paid continuous attention.

(3) Creep of the main girder

During the whole monitoring process, all displacement sensors of the main girder were in normal working condition, and all data fluctuated within a small range, with no data exceeding the limit; this indicates that the previous structural reinforcement measures were effective for controlling the main girder creep.

(4) Crack width

For the monitoring of crack width, all sensors also worked normally. However, the data of only one position were in a normal state, and the three other positions had data that exceeded the limit (listed in Table 6); this means that the bridge crack width should be the focus of continuous monitoring. Considering the influence of on-site construction, the data for changes in crack width are within the normal acceptable range. However, if the crack width continues to develop, appropriate measures should be taken to repair and restrain the development of these cracks.

(5) Settlement of the bridge piers

Due to the on-site construction under the bridge, the monitoring of pier settlement was delayed until 25 June 2018. From 25 June to 6 August 2018, the sensors at the settlement points of the pier were in normal working order. Based on the monitoring data, it was found that there were settlement development problems in two areas, but the limit value had not yet been exceeded. Considering that they may be affected by site construction, these two areas require continuous attention in the future. Fortunately, the data in other areas were stable, without any fluctuation or development trends.

Overall, based on the issues identified during the bridge monitoring process, the following recommendations are proposed for follow-up monitoring:

(1) As the strain levels of the W32# girder as well as W32-2# piers exceeded the limit and tended to increase gradually, as demonstrated in Figure 13a,c, respectively, it is recommended that the pier of W32-2# undergoes an appearance quality inspection, and that the damaged and exposed sections of the girder be repaired and the pier reinforced to ensure the structural integrity of the bridge.
(2) Due to the aging of the bridge and the occurrence of numerous subtle issues, it is suggested that continuous monitoring of the bridge be implemented to prevent the escalation of these problems. Particular attention should be paid to areas that slightly exceeded their limits and to the two piers (E32# and W37#) that are expected to exceed their limits soon.

## 5. Conclusions

In this work, the operational status of the targeted crossroad bridge was closely monitored by establishing a bridge monitoring system. Over the past few years, several inspections have been conducted on the crossroad bridge to identify its major issues. Based on the locations of the troublesome areas identified during the inspections, monitoring sensors were installed on the main girders and piers of the bridge to monitor the real-time changes in the structure. Meanwhile, the finite element model of the crossroad bridge was developed to perform stress analysis of the bridge during its operational stage. The strain levels of the main girders and piers, the deflection of the girders, and the settlement of piers were all calculated, and the resulting values were used as warning thresholds in the monitoring system. In the event that any of these values exceeded their respective warning thresholds, the corresponding areas were identified, and appropriate measures were taken promptly to ensure the structural safety of the crossroad bridge.

The monitoring process for the crossroad bridge lasted for 6 months, during which data on the strain and creep of the main beam, the strain and settlement of the piers, and the crack width of the bridge body were collected. Based on the monitoring data from February to August 2018, the real-time working state of the crossroad bridge was analyzed and evaluated. No abnormalities were found in the bridge displacement and pier settlement, and data changes were within the acceptable range due to the on-site construction environment near the structure. However, the strain-monitoring data of some girders (W13#, W32#, W35#, E26#, E28a, E29#) as well as some piers (W10#, W32-2#) exceeded the limits. In addition, the crack widths of piers W10# and W36-2# had noticeably expanded. Based on the monitoring data and the actual situation on site, it was determined that the appearance quality of pier W32-2# needed to be inspected and that the damaged as well as the exposed parts of the beam need to be repaired, with reinforcement of the pier at the same time, to ensure the integrity of the beam structure. For the bridge structures in the main girder areas that slightly exceeded the limit, or the two piers (E32#, W37#) that were about to exceed the limit, it is recommended that continuous attention be paid to them in the future. 

Through the practical application of SHM in the repaired and reinforced crossroad bridge structure, this study determined the actual operational status of the reinforced crossroad bridge through the analysis of the long-term monitoring data of Shenzhen crossroad bridge and put forward the corresponding countermeasures for some existing problems. These results can provide a valuable reference for future research and the practical application of SHM in crossroad bridges with similar bridge structures.

## Figures and Tables

**Table 1:** Locations of crack monitoring points.

No.	Location
1	Main girder: The outer web of bracket at the top of E11 split pier
2	Main girder: The outer web of bracket at top of W10# split pier
3	Pier: W34# pier column
4	Pier: W36-2# pier column

**Table 2:** Arrangement of settlement points.

No.	Location (Pier)
1	E4#
2	E5#
3	W3#
4	W4-1#
5	W37#
Datum Point 1	W4-2#
Datum Point 2	E32-1#

**Table 3:** Maximum and minimum moments of the main girder of the triple bridge span under a dead load.

Bridge Section	Maximum Moment (KN·m)	Minimum Moment (KN·m)
West No.9#	2.32893 × 10^4^	−4.27656 × 10^4^
West No.11#	4.67143 × 10^4^	−5.95503 × 10^4^
West No.6#	4.40943 × 10^4^	−5.96779 × 10^4^

**Table 4:** Maximum and minimum moments of the main girder of the triple bridge span under a live load.

Bridge Section	Maximum Moment (KN·m)	Minimum Moment (KN·m)
West No.9#	8.15062 × 10^3^	−6.80009 × 10^3^
West No.11#	1.27369 × 10^4^	−1.094710 × 10^4^
West No.6#	1.22862 × 10^4^	−1.104110 × 10^4^

**Table 5:** Warning values of each monitoring item based on FEM analysis.

Monitoring Items	Warning Values	Unit	Location
Strain of the main girder	100	με	W3#, W11#, W12#, W13#, W30#, W32#, W33#, W35#, E3#, E5#, E11#, E12#, E26#, E28a#, E29#
Strain of bridge piers	200	με	W32-2#, W34#, W2, W34#, W40-2#, W32-1, W32-2, W10, W12
Creep of the main girder	20	mm	W10, E11, W2, E29, W34, W7, E8, W5, E6, E2, W2, E25, W32-1,W32-2
Crack width	0.05	mm	E11#, W34#, W36-2#, W10#
Settlement of bridge piers	20	mm	W37#, E32-1#, W4-1#, W4-2#, W3#, E4#, E5#

**Table 6:** Monitoring results during the whole monitoring process.

Monitoring Items	Monitoring Results
Strain of the main girder	W13#, W32#, W35#, E26#, E28a#, E29#: Exceed the limit
Strain of bridge piers	W10#, W32-2#: Exceed the limit
Creep of the main girder	No abnormality
Crack width	W10#, W36-2#: Existing cracks show obvious expansion
Settlement of bridge piers	No abnormality

## Data Availability

All data included in this study are available when required.
